# P-361. Improving ART Adherence and HIV Care Engagement Through Multi-Faceted Interventions

**DOI:** 10.1093/ofid/ofaf695.579

**Published:** 2026-01-11

**Authors:** Lisa B Hightow-Weidman, Tamar Sapir, Leah Molloy, Laura Simone, Chris Napolitan, Jeffrey D Carter, Melissa Rodriguez, Chelsie Chadha, Kelly E Pillinger

**Affiliations:** Florida State University, Tallahassee, Florida; Synchronyx, Boca Raton, Florida; PRIME Education, Brighton, Michigan; PRIME Education, LLC, Fort Lauderdale, Florida; PRIME Education, Brighton, Michigan; PRIME Education, LLC, Fort Lauderdale, Florida; PRIME Education, Brighton, Michigan; PRIME Education, Brighton, Michigan; PRIME Education, Brighton, Michigan

## Abstract

**Background:**

Adherence to antiretroviral therapy (ART) is an important driver of long-term outcomes, yet barriers can make this challenging. This quality improvement initiative aimed to improve engagement and adherence among people with HIV (PWH) through patient-provider collaborative learning sessions (CLS) and by piloting a remote medication companion tool, Tappt® Health.

**Methods:**

From July to Dec 2024, healthcare professionals (HCPs) at 4 clinics participated in audit-feedback (A/F) sessions to create action plans to improve adherence and held CLS to engage PWH. Surveys were given to PWH and HCPs pre- and post-session. Beginning in Jan 2025, two clinics piloted the Tappt® Health app where PWH tracked adherence and completed surveys over three months. HIV teams were alerted if PWH reported nonadherence or barriers.

**Results:**

HCPs (N = 50) who attended A/F sessions reported 40% of their clients living with HIV experienced adherence challenges. Following the session, HCP confidence in asking PWH about adherence numerically increased from 56% to 70% (p = 0.17), with 55% planning to implement increased ART education. CLS were attended by 70 PWH (mean age 45 years; 58% Hispanic/Latinx; 49% living with HIV ≥ 10 years). Non-adherence was high, with 45% reporting they had taken their ART 2 days or less in the past week. Following the CLS, PWHs’ confidence in taking ART as prescribed increased from 67% to 91% (p = 0.003). Top adherence challenges were forgetting (36%) or a busy schedule (34%) with 51% planning to discuss other ART options with their HCP after the CLS. To date, 47 PWH enrolled in Tappt®, with 25 engaged users. Of engaged users, there was a 73% adherence rate. Alerts to HCPs were triggered due to reported social determinants of health (SDOH) (60% of users) and if < 80% adherent in last 28 days (36% of users). Users reporting at least 1 SDOH (n = 15) had a higher average adherence rate than those without any (n = 10) (87% vs. 52%, respectively) likely driven by HCP actions in response to the alerts (53% 1 SDOH vs. 20% no SDOH).

**Conclusion:**

This QI project improved PWH confidence in taking ART and showed a trend toward improved HCP confidence in asking about adherence. Interestingly, adherence rates in PWH with SDOH were higher, likely driven by alerts sent to HCPs that resulted in increased support between clinic appointments.

**Disclosures:**

Tamar Sapir, PhD, Synchronyx: Board Member|Synchronyx: Ownership Interest

